# Prospective open-label study of add-on and monotherapy topiramate in civilians with chronic nonhallucinatory posttraumatic stress disorder

**DOI:** 10.1186/1471-244X-4-24

**Published:** 2004-08-18

**Authors:** Jeffrey L Berlant

**Affiliations:** 1 Private practice, Boise, ID, USA

## Abstract

**Background:**

In order to confirm therapeutic effects of topiramate on posttraumatic stress disorder (PTSD) observed in a prior study, a new prospective, open-label study was conducted to examine acute responses in chronic, nonhallucinatory PTSD.

**Methods:**

Thirty-three consecutive newly recruited civilian adult outpatients (mean age 46 years, 85% female) with DSM-IV-diagnosed chronic PTSD, excluding those with concurrent auditory or visual hallucinations, received topiramate either as monotherapy (n = 5) or augmentation (n = 28). The primary measure was a change in the PTSD Checklist-Civilian Version (PCL-C) score from baseline to 4 weeks, with response defined as a ≥ 30% reduction of PTSD symptoms.

**Results:**

For those taking the PCL-C at both baseline and week 4 (n = 30), total symptoms declined by 49% at week 4 (paired *t*-test, *P *< 0.001) with similar subscale reductions for reexperiencing, avoidance/numbing, and hyperarousal symptoms. The response rate at week 4 was 77%. Age, sex, bipolar comorbidity, age at onset of PTSD, duration of symptoms, severity of baseline PCL-C score, and monotherapy versus add-on medication administration did not predict reduction in PTSD symptoms. Median time to full response was 9 days and median dosage was 50 mg/day.

**Conclusions:**

Promising open-label findings in a new sample converge with findings of a previous study. The use of topiramate for treatment of chronic PTSD, at least in civilians, warrants controlled clinical trials.

## Background

Posttraumatic stress disorder (PTSD) is a difficult-to-treat condition that over a lifetime affects approximately 10% of the general population [1]. The condition develops after traumatic events such as combat, terror activities, disaster, or rape, and has 3 main features: (1) reexperiencing the trauma through recollection, dreams, and reliving, (2) avoidance of thoughts, activities, and emotions associated with the trauma, and (3) hyperarousal [2]. PTSD is usually a chronic disorder, with one third of patients displaying symptoms for ≥ 10 years after experiencing the traumatic event [3,4]. Generally the response to pharmacotherapy has been poor, with many patients completely unresponsive and others only marginally responsive [5]. Some tricyclic antidepressants, monoamine oxidase inhibitors, and selective serotonin reuptake inhibitors have demonstrated efficacy in double-blind trials [3]. The complex neurobiology of PTSD involves a number of systems, including dopaminergic, serotonergic, sympathetic, hypothalamic-pituitary-adrenal, and various anatomic regions of the amygdala and other parts of the limbic system [3]. It has been suggested that after traumatic events limbic nuclei may become kindled or abnormally sensitized [6], leading to increased susceptibility for psychic and physical arousal and psychiatric disturbance [2]. Because of the suggested involvement of the kindling phenomenon, several anticonvulsants have been assessed in the treatment of PTSD, including carbamazepine, valproate, lamotrigine, gabapentin, tiagabine, and topiramate [3].

Topiramate has a broad spectrum of pharmacologic properties, including Na^+ ^channel blockade [7-11], inhibition of some high voltage-activated Ca^2+ ^channels [12], enhanced γ-aminobutyric acid (GABA) neuroinhibition at novel GABA_A _receptors [13,14], glutamate inhibition at kainate and α-amino-3-hydroxy-5-methyl-4-isoxazole propionic acid (AMPA) receptors [15,16], and promotion of protein phosphorylation of neuronal conductance channels [15]. These properties, together with inhibitory activity in animal kindling models [17,18], suggest that topiramate may have therapeutic potential in PTSD.

Treatment with topiramate has been reported to improve reexperiencing symptoms associated with civilian PTSD [6]. Results from that study, which included a number of patients who were classified as treatment resistant, suggested that topiramate fully or partially suppressed both intrusions (distressing recollections or nonhallucinatory flashbacks) and nightmares, if present, in 89% of patients with nonhallucinatory PTSD.

Although the DSM-IV definition of PTSD lists hallucinations among reexperiencing symptoms [19], because a few patients displayed varying degrees of impaired reality testing of hallucinations, it was difficult to determine if hallucinations were due to PTSD, to an independent psychotic disorder, or, in some individuals, to both. Although psychotic variants of PTSD have been reported in as many as 40% of veterans with chronic combat-associated PTSD without evidence of a primary psychotic disorder [20,21], it is also possible for patients with psychotic disorders to have comorbid PTSD, leaving questions about whether specific hallucinations should be attributable to the PTSD reexperiencing cluster or to a psychotic disorder. Because the prior study found a more robust effect in the group of PTSD patients without hallucinations [6], it was decided to focus further on the core group of PTSD patients without ambiguous symptoms confounded by the possible presence of independent psychotic disorders.

Based on these earlier results of topiramate in PTSD, a fully prospective open-label study was begun to see whether the previously observed signal of a therapeutic benefit of topiramate for chronic civilian PTSD could be confirmed. None of the patients reported in the earlier study were included in the present study. In order to address methodological limitations of the prior study, the current study is modified in 4 major ways: (1) it excludes patients with hallucinations, (2) it limits the study to 12 weeks, (3) it prospectively employs the PTSD Checklist-Civilian Version (PCL-C) as a validated clinical assessment instrument in all patients, and (4) it determines a clinical response rate as of week 4, using a conventional, predetermined definition for positive response as a ≥ 30% improvement in a standardized clinical rating scale.

## Methods

This sample included all consecutive adults (n = 33) meeting the DSM-IV criteria for chronic civilian PTSD in an outpatient private practice who started open-label topiramate between January 2000 and November 2002 in the course of clinical care. The study excluded patients with concurrent auditory or visual hallucinations associated with either PTSD or a possible psychotic disorder. Topiramate, administered either as monotherapy (n = 5) or added to the patients' existing therapeutic regimen (n = 28) (Table 1), was initiated at a starting dosage of 12.5 to 25 mg/day and increased in 25- to 50-mg/day increments every 3 to 4 days as tolerated until a clinical response was achieved.

**Table 1:** Patient characteristics

	All subjects N = 33
Mean age, years ± SD	46 ± 6.5
Range	29–55
Sex, %	
Women	85
Men	15
Mean age at onset of PTSD, years ± SD	29 ± 15
Range	2–53
Mean duration of PTSD history, years ± SD	18 ± 15
Range	0–46
Patients with comorbid disorders, N (%)	
Bipolar disorder	10 (30)
Major depressive disorder	21 (64)
Substance abuse	
Current	3 (9)
In past	5 (15)

SD =standard deviation; PTSD = posttraumatic stress disorder.

The study was performed according to the Declaration of Helsinki. Each participant provided verbal informed consent based on disclosure of off-label usage, the availability of alternative medications for PTSD such as sertraline and paroxetine, and expected adverse events and risks. Verbal consent was deemed appropriate and sufficient because of the nonexperimental nature of the treatment. Because this was a descriptive study using aggregate data gathered in the course of treating patients in clinical practice for their own benefit and not an experimental design, there was no protocol that required Institutional Review Board approval. Patients were asked to measure their symptoms using standardized clinical instruments already routinely used as a part of individual care. The study included no features of clinical studies, such as randomization, concealment of treatment, or surrender of patient decision-making rights, as an integral part of conducting the study.

The primary outcome was a change in PTSD symptoms calculated from change in PCL-C score from baseline through 4 weeks of treatment [22]. The PCL-C, a validated self-report instrument, strongly correlates (*k*~0.90) with the Clinician-Administered PTSD Scale (CAPS), a structured clinical interview widely used in PTSD studies [23]. The PCL-C is scored on a Likert scale of 1 to 5 (1 = not at all; 5 = extremely) for each of 17 items that cover all 3 symptom clusters (B, reexperiencing; C, avoidance/numbing; D, hyperarousal), yielding a score range of 17 to 85. A total score of ≥ 50 meets the threshold for active PTSD. In accordance with conventional usage, a 30% reduction in clinical symptoms of PTSD was defined as a positive clinical response. The secondary measures included were Cluster B symptoms of reexperiencing of trauma, including trauma-related intrusions and nightmares. Secondary measures were limited to Cluster B symptoms because of the practical difficulty patients in clinical practice can have linking a symptom (such as avoidance, numbing, hyperarousal, insomnia, or startling) specifically to a trauma as opposed to some other source, as required by the DSM-IV definitions. Also, Cluster B symptoms appeared to be among the most distressing and salient for many patients, thereby making tracking these distinctive symptoms comparatively easy.

Data on patient demographics, concomitant medication, comorbidities, primary trauma, and duration of PTSD were collected at baseline. Assessments were made through clinical interviews over a 12-week period. Cessation of reexperiencing symptoms was considered partial if subjects reported a definite reduction in intensity and frequency of nightmares or intrusive recollections or flashbacks. Full cessation was defined as complete elimination of nightmares and intrusions for a sustained period. Adverse effects were recorded if they resulted in treatment discontinuation.

Statistical analyses were performed using Jandel SigmaStat^® ^Version 2.0 (Jandel Corporation, San Rafael, CA, USA). A single paired *t*-test of before and after total PCL-C scores was used to analyze the data. Subscale scores for PCL-C Clusters B, C, and D were also evaluated.

## Results

### Patient characteristics

Baseline patient characteristics and concomitantly administered medications are listed in Tables 1 and 2, respectively. All monotherapy patients were in the nonbipolar subgroup. Primary traumas for which symptoms were reported are detailed in Table 3. Of these, 9 were of childhood onset, with 4 involving childhood physical assault, 3 unwanted sex or sexual assault, and 2 sudden unexpected or violent deaths.

**Table 2:** Concomitant medications

**Medication type**	**Number of patients (N = 33)**
SSRIs	12
Benzodiazepines	10
Stimulants	6
Atypical neuroleptics	3
Gabapentin	3
Lamotrigine	5
Mirtazapine	3
Venlafaxine	3
Verapamil	1
Other	8
Monotherapy	5

SSRIs = selective serotonin reuptake inhibitors.

**Table 3:** Primary traumas

**Trauma type**	**Number of patients, N = 33**
Unwanted sex	11
Physical assault	6
Sudden violent death	4
Sudden unexpected death of loved one	3
Sexual assault	2
Fire/explosion	1
Weapon assault	1
Combat	1
Life-threatening illness	1
Other	3

### Primary measures

#### Reduction in PCL-C symptoms

**Figure 1:**
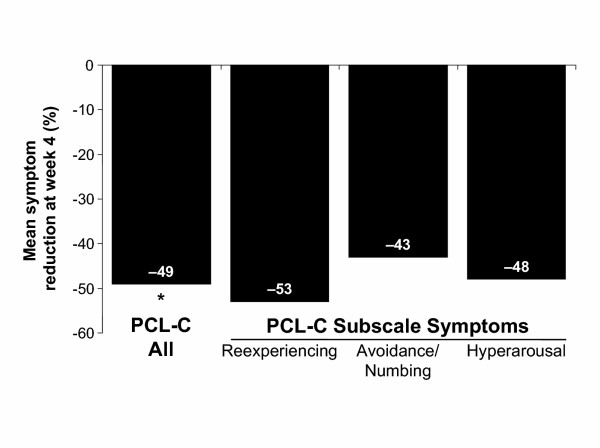
**Mean percentage symptom reduction at week 4. **Symptom reduction in those 30 subjects who completed a PCL-C at baseline and after 4 weeks. *Paired *t*-test, *P *< 0.001 versus baseline. PTSD = posttraumatic stress disorder; PCL-C = PTSD Checklist-Civilian Version.

Ninety-one percent of patients (30/33) with baseline PCL-C measurements completed a PCL-C at week 4. In those patients, the total score was 62.6 at baseline and 40.3 at week 4. In addition, the scores for reexperiencing, avoidance, and hyperarousal clusters were 18.2, 25.1, and 19.2 at baseline and 11.2, 17.2, and 12.3 at week 4, respectively. To calculate percentage reduction in PTSD symptoms, to correct for an absence of symptoms being scored as "1" for each item, the change in score between baseline and week 4 was divided by the baseline score minus the minimum score for no symptoms. Therefore, the change in total symptoms was calculated as (baseline – week 4)/(baseline – 17). Total symptoms declined significantly (49%) by week 4 (paired *t*-test, *P *< 0.001). In addition, subscale symptoms for reexperiencing (Cluster B), avoidance/numbing (Cluster C), and hyperarousal (Cluster D) decreased by 53%, 43%, and 48%, respectively, from baseline (Figure 1). By the end of week 4, 70% of all entrants in the study (n = 33) and 77% of those with both baseline and week 4 PCL-C scores (n = 30) were positive responders, defined as a ≥ 30% improvement in symptoms.

#### Predictors of reduction in PCL-C symptom improvement

Comparisons for bipolar versus nonbipolar patients (50% versus 49%, analysis of variance [ANOVA]), duration of symptoms (r = 0.37), age at onset of PTSD (r = -0.36), severity of baseline PCL-C score (r = 0.02), age (r = -0.02), female sex (49% versus 50%, ANOVA), and monotherapy versus add-on medication administration (53% versus 48%, ANOVA) found none significantly associated with reduction in PCL-C symptoms.

### Secondary measures

#### Results for combined suppression of nightmares and intrusions are shown in Table 4

**Table 4:** Secondary efficacy measures

**Combined suppression of nightmares and intrusions**	
Responder status, n (%)	
Full	26/33 (79%)
Partial	3/33 (9%)
None	4/33 (12%)
Mean time to response, days ± SD, (range)	
Full response (n = 25)	15 ± 18 (1–83)
Partial response (n = 17)	11 ± 13 (2–46)
Median time to full response, days	9
Median time to partial response, days	5
Mean dosage at time of response, mg/day	
Full response	60 ± 47
Partial response	32 ± 15
Median dosage at time of response, mg/day	
Full response	50
Partial response	25
Modal daily dosage for full response, mg/day	25
% of patients in the 12.5–50-mg/day range	65
Modal dosage at time of partial response, mg/day	25
% of patients in the 12.5–50-mg/day range	100
**Improvements in nightmares**	
Full cessation of nightmares	17/18 (94%)
**Improvements in intrusions**	
Full cessation of intrusions	26/33 (79%)
Partial improvement	3/33 (9%)
No improvement	4/33 (12%)

SD = standard deviation.

Of the 33 patients who entered the study, 79% fully ceased having reexperiencing symptoms with a mean time to full cessation of 15 days. The onset of full cessation for all patients occurred at ≤ 200 mg/day and the onset of partial cessation for all patients at ≤ 50 mg/day. Ninety-four percent of patients with nightmares and 79% of patients with intrusions at baseline reported full cessation at the end of week 4.

### Discontinuations

#### Serious adverse events

There were no serious adverse events during this study. Of the 33 patients who entered the study, 7 (21%) discontinued due to adverse events and 5 (15%) for other reasons. The single most common adverse event resulting in discontinuation was intolerable nervous system overstimulation (panic/nervousness/overstimulation/ shakiness), reported by 3 patients. Other adverse events resulting in discontinuation included clumsiness (n = 2), cognitive impairment (n = 1), and severe headache (n = 1). Additional reasons for discontinuation included personal choice (n = 1) and lack of relapse of PTSD symptoms after interrupting medication (n = 4). Of the 12 patients that discontinued, 54% (n = 7) fully ceased having reexperiencing symptoms of nightmares/intrusions and 15% (n = 2) partially ceased having reexperiencing symptoms of nightmares/intrusions. The median time to termination was 25.5 days (range, 4–77).

#### Predictors of discontinuation

There were no statistically significant predictors of panic-like adverse events resulting in discontinuation from the study (age, sex, symptom duration, age at onset, bipolarity, substance abuse, baseline severity of illness), although the coadministration of a benzodiazepine nearly reached significance (Fisher exact test, *P *= 0.05). No monotherapy patients discontinued because of overstimulation/panic-like responses, so the possibility of drug interactions or conditions such as comorbid panic disorder contributing to this reaction should be considered in future investigations.

## Discussion

Results of this prospective open-label study suggest that the use of topiramate for the treatment of chronic PTSD in civilians is indeed promising and warrants controlled clinical trials. Topiramate was able to improve symptoms of reexperiencing, avoidance, and hyperarousal by 49% overall, compared with baseline, and decreased intrusions in 94% of patients and nightmares in 79% of those with this symptom.

Although there are numerous other reports describing the use of antiepileptic agents for the treatment of PTSD, most are case reports and case series, and some lack the use of standardized PTSD scales. The first published trials using anticonvulsants date back almost 20 years, when a 5-week trial of carbamazepine in 10 combat veterans produced a 36% reduction in a nonvalidated interviewer-scored instrument [24]. Studies on adjunctive therapy with valproate reported improved global scores in avoidance and hyperarousal, but not reexperiencing symptoms in veterans over a 14-month period [25], whereas a subsequent 8-week open-label trial reported a small, 17% reduction in CAPS score in 13 subjects with significant reductions in all 3 cluster criteria [26]. Two studies in which valproate was used as monotherapy reported varying results, with one showing a 30% reduction in total CAPS scores in 28 veterans [27], whereas the other in 10 civilian patients with non-combat-related PTSD showed no improvement [28].The only double-blind placebo-controlled trial evaluating an anticonvulsant in PTSD was conducted with lamotrigine [29]. Overall, after 10 weeks of treatment, 5 of 10 subjects were characterized as responders, compared with 1 of 4 subjects receiving placebo. However, a last observation carried forward analysis revealed only a 23% decrease in mean pretreatment scores with lamotrigine, compared with 20% in the placebo group. A retrospective clinical series of adjunctive gabapentin therapy in 30 consecutive patients with PTSD assessed improvement of target symptoms such as nightmares, insomnia, and irritability. It was reported that the majority of patients (77%) showed moderate or greater improvement in duration of sleep, and most notably a decrease in the frequency of nightmares [30]. These studies highlight the need for double-blind placebo-controlled trials using standardized and validated questionnaires.

It is encouraging that the results of this current study with topiramate were consistent with the earlier, preliminary data obtained in patients with PTSD. Percentage responders (77% versus 75%), median time to full response (9 d versus 8 d), suppression of intrusions and nightmares (88% versus 89%), and discontinuation rate (39% versus 32%) were all similar. Overall, the open-label experience with topiramate, based on patient statements and self-rating reports, suggests that the drug may have a rapid rate of response with limited risk of clinically significant adverse events and no evidence of tolerance developing over time [6,31]. Moreover, from topiramate studies in epilepsy [Topamax prescribing information] it is known that the drug is not associated with the cardiac, pancreatic, and hematologic toxicity found with valproate or carbamazepine [3]. In addition, topiramate is not associated with weight gain, a side effect reported with valproate that can predispose patients to diabetes mellitus [32]. Topiramate also offers potential clinical advantages over serotonin reuptake inhibitors (SSRIs) because it does not appear to destabilize mood in patients with comorbid bipolar disorder and is relatively devoid of common adverse effects of SSRIs, such as sexual dysfunction, weight gain with chronic use, or sedation when used as monotherapy.

Limitations of the present study include: (1) the absence of structured assessment data following week 4 to test the maintenance of response over time, (2) use of self-report instruments that, although validated and correlated with results of structured clinical interviews, may be less accurate than structured interviews, and (3) the absence of clinical assessment scores at the time patients discontinued the trial. From a broader perspective, the greatest limitation of this study is characteristic of all open trials: the lack of standard features of clinical trials such as placebo controls, randomization, and blinding of raters.

## Conclusions

Fundamentally, the findings of current and prior topiramate studies are convergent and consistently signal a potentially beneficial therapeutic effect for all 3 criteria clusters of chronic PTSD in civilian adults. In both studies, there is a positive response in a large proportion of patients, at dosages considerably below the usual anticonvulsant dosage levels of 200 to 600 mg/day, and a rapid onset. The effect appears independent of comorbidity with bipolar disorder, age, sex, duration of symptoms, baseline severity of illness, or administration alone or with other psychotropic medications. The purpose of this study was to prospectively test the results of an earlier open-label trial in a new sample; however, the limitations of those open studies will have to be addressed in double-blind, placebo-controlled studies, which are currently in progress.

## Competing interests

The author is a consultant to and a participant in the Speaker's Bureau for Ortho-McNeil Pharmaceutical, Inc. He also holds a licensing agreement with Ortho-McNeil based on issuance of a United States Government patent for topiramate therapy of PTSD.

## Author's contributions

JLB conceived of the study and its design, coordination, statistical analysis, and drafted the manuscript.

## Pre-publication history

The pre-publication history for this paper can be accessed here:



## References

[B1] Breslau N (2001). The epidemiology of posttraumatic stress disorder: what is the extent of the problem?. J Clin Psychiatry.

[B2] van der Kolk BA (1997). The psychobiology of posttraumatic stress disorder. J Clin Psychiatry.

[B3] Iancu I, Rosen Y, Moshe K (2002). Antiepileptic drugs in posttraumatic stress disorder. Clin Neuropharmacol.

[B4] Berlant J (2003). Antiepileptic treatment of posttraumatic stress disorder. Primary Psychiatry.

[B5] Davidson JR (1997). Biological therapies for posttraumatic stress disorder: an overview. J Clin Psychiatry.

[B6] Berlant J, van Kammen DP (2002). Open-label topiramate as primary or adjunctive therapy in chronic civilian posttraumatic stress disorder: a preliminary report. J Clin Psychiatry.

[B7] DeLorenzo RJ, Sombati S, Coulter DA (2000). Effects of topiramate on sustained repetitive firing and spontaneous recurrent seizure discharges in cultured hippocampal neurons. Epilepsia.

[B8] Zona C, Ciotti MT, Avoli M (1997). Topiramate attenuates voltage-gated sodium currents in rat cerebellar granule cells. Neurosci Lett.

[B9] McLean MJ, Bukhari AA, Wamil AW (2000). Effects of topiramate on sodium-dependent action-potential firing by mouse spinal cord neurons in cell culture. Epilepsia.

[B10] Taverna S, Sancini G, Mantegazza M, Franceschetti S, Avanzini G (1999). Inhibition of transient and persistent Na+ current fractions by the new anticonvulsant topiramate. J Pharmacol Exp Ther.

[B11] Wu SP, Tsai JJ, Gean PW (1998). Frequency-dependent inhibition of neuronal activity by topiramate in rat hippocampal slices. Br J Pharmacol.

[B12] Zhang X, Velumian AA, Jones OT, Carlen PL (2000). Modulation of high-voltage-activated calcium channels in dentate granule cells by topiramate. Epilepsia.

[B13] White HS, Brown SD, Woodhead JH, Skeen GA, Wolf HH (1997). Topiramate enhances GABA-mediated chloride flux and GABA-evoked chloride currents in murine brain neurons and increases seizure threshold. Epilepsy Res.

[B14] White HS, Brown SD, Woodhead JH, Skeen GA, Wolf HH (2000). Topiramate modulates GABA-evoked currents in murine cortical neurons by a nonbenzodiazepine mechanism. Epilepsia.

[B15] Gibbs JW, Sombati S, DeLorenzo RJ, Coulter DA (2000). Cellular actions of topiramate: blockade of kainate-evoked inward currents in cultured hippocampal neurons. Epilepsia.

[B16] Skradski S, White HS (2000). Topiramate blocks kainate-evoked cobalt influx into cultured neurons. Epilepsia.

[B17] Wauquier A, Zhou S (1996). Topiramate: a potent anticonvulsant in the amygdala-kindled rat. Epilepsy Res.

[B18] Amano K, Hamada K, Yagi K, Seino M (1998). Antiepileptic effects of topiramate on amygdaloid kindling in rats. Epilepsy Res.

[B19] (1994). DSM-IV: Diagnostic and Statistical Manual of Mental Disorders.

[B20] Hamner MB (1997). Psychotic features and combat-associated PTSD. Depress Anxiety.

[B21] Hamner MB, Frueh BC, Ulmer HG, Arana GW (1999). Psychotic features and illness severity in combat veterans with chronic posttraumatic stress disorder. Biol Psychiatry.

[B22] Blanchard EB, Jones-Alexander J, Buckley TC, Forneris CA (1996). Psychometric properties of the PTSD Checklist (PCL). Behav Res Ther.

[B23] Weathers FW, Keane TM, Davidson JR (2001). Clinician-administered PTSD scale: a review of the first ten years of research. Depress Anxiety.

[B24] Lipper S, Davidson JR, Grady TA, Edinger JD, Hammett EB, Mahorney SL, Cavenar JO (1986). Preliminary study of carbamazepine in post-traumatic stress disorder. Psychosomatics.

[B25] Fesler FA (1991). Valproate in combat-related posttraumatic stress disorder. J Clin Psychiatry.

[B26] Clark RD, Canive JM, Calais LA, Qualls CR, Tuason VB (1999). Divalproex in posttraumatic stress disorder: an open-label clinical trial. J Trauma Stress.

[B27] Petty F, Davis LL, Nugent AL, Kramer GL, Teten A, Schmitt A, Stone RC (2002). Valproate therapy for chronic, combat-induced posttraumatic stress disorder. J Clin Psychopharmacol.

[B28] Otte C, Wiedemann K, Yassouridis A, Kellner M (2004). Valproate monotherapy in the treatment of civilian patients with non-combat-related posttraumatic stress disorder: an open-label study. J Clin Psychopharmacol.

[B29] Hertzberg MA, Butterfield MI, Feldman ME, Beckham JC, Sutherland SM, Connor KM, Davidson JR (1999). A preliminary study of lamotrigine for the treatment of posttraumatic stress disorder. Biol Psychiatry.

[B30] Hamner MB, Brodrick PS, Labbate LA (2001). Gabapentin in PTSD: a retrospective, clinical series of adjunctive therapy. Ann Clin Psychiatry.

[B31] Berlant JL (2001). Topiramate in posttraumatic stress disorder: preliminary clinical observations. J Clin Psychiatry.

[B32] Corman CL, Leung NM, Guberman AH (1997). Weight gain in epileptic patients during treatment with valproic acid: a retrospective study. Can J Neurol Sci.

